# Impacts of the COVID-19 pandemic on access to HIV and reproductive health care among women living with HIV (WLHIV) in Western Kenya: A mixed methods analysis

**DOI:** 10.3389/fgwh.2022.943641

**Published:** 2022-12-12

**Authors:** Caitlin Bernard, Shukri A. Hassan, John Humphrey, Julie Thorne, Mercy Maina, Beatrice Jakait, Evelyn Brown, Nashon Yongo, Caroline Kerich, Sammy Changwony, Shirley Ru W. Qian, Andrea J. Scallon, Sarah A. Komanapalli, Leslie A Enane, Patrick Oyaro, Lisa L. Abuogi, Kara Wools-Kaloustian, Rena C. Patel

**Affiliations:** ^1^ Department of Obstetrics and Gynecology, Indiana University School of Medicine, Indianapolis, IN, United States; ^2^ Department of Medicine, University of Washington, Seattle, WA, United States; ^3^ Department of Medicine, Indiana University School of Medicine, Indianapolis, IN, United States; ^4^ Department of Obstetrics and Gynecology, University of Toronto, Toronto, ON, Canada; ^5^ Academic Model Providing Access to Healthcare (AMPATH), Eldoret, Kenya; ^6^ UW Kenya, Nairobi, Kenya; ^7^ Department of Public Health, University of Washington, Seattle, WA, United States; ^8^ Jackson School of International Studies, University of Washington, Seattle, WA, United States; ^9^ Indiana University School of Medicine, Indianapolis, IN, United States; ^10^ Department of Pediatrics, Indiana University School of Medicine, Indianapolis, IN, United States; ^11^ Health Innovations Kenya (HIK), Kisumu, Kenya; ^12^ Department of Pediatrics, University of Colorado, Denver, CO, United States

**Keywords:** COVID-19, family planning (FP), pregnancy, HIV, antiretroviral (ARV) therapy

## Abstract

**Introduction:**

The COVID-19 pandemic has impacted access to health services. Our objective was to understand the pandemic's impact on access to HIV, pregnancy, and family planning (FP) care among women living with HIV (WLHIV).

**Methods:**

Data were collected after June 2020, when questions about the pandemic were added to two ongoing mixed methods studies using telephone surveys and in-depth interviews among WLHIV in western Kenya. The *Chaguo Langu (CL)* study includes primarily non-pregnant WLHIV receiving HIV care at 55 facilities supported by AMPATH and the *Opt4Mamas* study includes pregnant WLHIV receiving antenatal care at five facilities supported by FACES. Our outcomes were self-reported increased difficulty refilling medication, accessing care, and managing FP during the pandemic. We summarized descriptive data and utilized multivariable logistic regression to evaluate predictors of difficulty refilling medication and accessing care. We qualitatively analyzed the interviews using inductive coding with thematic analysis.

**Results:**

We analyzed 1,402 surveys and 15 in-depth interviews. Many (32%) *CL* participants reported greater difficulty refilling medications and a minority (14%) reported greater difficulty accessing HIV care during the pandemic. Most (99%) *Opt4Mamas* participants reported no difficulty refilling medications or accessing HIV/pregnancy care. Among the *CL* participants, older women were *less* likely (aOR = 0.95, 95% CI: 0.92–0.98) and women with more children were *more* likely (aOR = 1.13, 95% CI: 1.00–1.28) to report difficulty refilling medications. Only 2% of *CL* participants reported greater difficulty managing FP and most (95%) reported no change in likelihood of using FP or desire to get pregnant. Qualitative analysis revealed three major themes: (1) adverse organizational/economic implications of the pandemic, (2) increased importance of pregnancy prevention during the pandemic, and (3) fear of contracting COVID-19.

**Discussion:**

The two unique participant groups included in our study encountered overlapping problems during the COVID-19 epidemic. Access to HIV services and antiretrovirals was interrupted for a large proportion of non-pregnant WLHIV in western Kenya, but access to pregnancy/family planning care was less affected in our cohort. Innovative solutions are needed to ensure HIV and reproductive health outcomes do not worsen during the ongoing pandemic.

## 1. Introduction

Since its identification in December 2019, SARS-CoV-2, the virus that causes COVID-19, has spread globally and has been declared a pandemic by the World Health Organization (WHO) (1). Countries around the world have initiated varying local and national policies that modified operations of healthcare and other services to accommodate travel restrictions, social distancing recommendations, and curfews. Globally, access to healthcare services, commodities, and pharmaceuticals has been variably disrupted, depending on local factors (2, 3). In some places and at some times, supply chain disruptions have occurred, leading to stock-outs of antiretrovirals (ARVs) and contraceptive commodities, among other life-saving treatment (3). Healthcare workers have been redirected to acute care needs, and lack of personal protective equipment and social distancing measures led to a reduction in service availability and clinic closures (4–6). Additionally, fear of infection and economic distress has hindered many from seeking needed healthcare, and healthcare utilization patterns have shifted dramatically during the pandemic (7).

Reproductive health (RH) services, including pregnancy and family planning (FP) care, have been especially affected, with concerns for resulting increases in unintended pregnancy, unsafe abortion, and maternal and neonatal mortality (8–11). For instance, the United Nations Population Fund estimates that the COVID-19 pandemic disrupted contraceptive use for up to 12 million women, resulting in 1.4 million unintended pregnancies in 2020 across 115 low- and middle-income countries (12). Similarly, there are reports of interruption in HIV testing, antiretroviral therapy (ART) initiation, and ART access for people living with HIV (PLHIV), increasing risks of HIV-associated mortality (5, 11, 13). Women and PLHIV in particular may be reluctant or unable to access care given their need to care for vulnerable family members and their own immunocompromised state. Pregnant women may have delayed seeking perinatal care due to the possibility of contracting COVID-19 (14). Such impacts on care access will likely have significant consequences for maternal and neonatal mortality as well as mortality from preventable diseases and complications, particularly for WLHIV (15, 16).

In order to understand the impact of the COVID-19 pandemic on the ability of WLHIV to access HIV and RH care, we leveraged our existing mixed-methods studies, named *Chaguo Langu—My Choice* and *Opt4Mamas*, to explore these questions among WLHIV engaged in care at public health facilities in western Kenya.

## 2. Materials and methods

### 2.1. Policy context

The Kenyan Ministry of Health (MOH) confirmed the first case of COVID-19 in Nairobi on March 12, 2020, and subsequently the Kenyan people experienced 338,379 confirmed cases of COVID-19 and 5,675 deaths, as of September 2022 (17). Soon after, the MOH put in place precautions and guidelines, including quarantining and social distancing, observing basic hygiene practices, mandatory face masks, and travel restrictions, including restricting local public transportation vehicles to operate at half-capacity (18). These restrictions not only disrupted public transportation operations, but also increased transportation costs and exacerbated existing barriers to transportation and access to care (19).

In response to the COVID-19 pandemic, the MOH and HIV care implementing partners, including the Academic Model for Providing Access To Healthcare (AMPATH) and Family AIDS Care and Education Services (FACES), continued to provide HIV and RH care services both in person and *via* telehealth services, with provision of multi-month refills of ARVs and increased community-based ARV delivery to decrease the need for clinic visits, when possible. Despite these interventions, service interruptions at many smaller facilities as well as pharmacy and clinic stock-outs did occur, particularly of ARVs and related medications and FP commodities.

### 2.2. Study design

This secondary analysis synthesizes data collected from two mixed methods studies: (1) *Chaguo Langu*, and (2) *Opt4Mamas*. We employed a mixed methods approach called concurrent triangulation design (20), in which we incorporated qualitative data from in-depth interviews to better understand and offer more context for the findings in the quantitative surveys. We chose to include both studies in our analysis because they are based in care systems that are both anchored in the public health care system and held accountable to the same Ministry of Health-led monitoring and evaluation system, and the ART and FP supplies are obtained *via* the same supply chain. We hypothesize that, as a result of living and accessing care in comparable settings, the women in both studies will have the same difficulties during the COVID-19 pandemic.

*Chaguo Langu* (“*My choice*” in Kiswahili) is a mixed methods study of WLHIV exposed to dolutegravir (DTG) as part of an ART regimen and enrolled in care at public health facilities in western Kenya supported by AMPATH, with its largest facility in Eldoret, Uasin Gishu County (21). This study included: (1) a retrospective cohort study of prospectively-collected electronic medical record (EMR) data for WLHIV exposed to DTG, (2) telephone surveys with a subset of this population, and (3) in-depth interviews with a subset of telephone survey participants.

The *Opt4Mamas* study is a prospective, mixed methods cohort study of pregnant WLHIV receiving antenatal care in one of the five public facilities in Kisumu County, Kenya supported by FACES and the International Center for AIDS Care and Treatment Program (ICAP) who were enrolled between March and November 2019 and followed until 6 months postpartum through August 2021. During the COVID-19 pandemic, study follow-up visits were performed *via* telephone.

Each study was approved by ethics review boards, including the Moi Teaching and Referral Hospital/Moi University Institutional Research and Ethics Committee and the Indiana University Institutional Review Board for *Chaguo Langu* and African Medical and Research Foundation (AMREF) for *Opt4Mamas* in Kenya, and the Human Subjects Division at the University of Washington for both. Only de-identified data were merged between the two studies and made accessible to investigators from each study.

### 2.3. Cohort selection

WLHIV were included in the *Chaguo Langu* study if they were: (1) enrolled in HIV care at an AMPATH-supported facility, (2) 15–49 years of age (inclusive) on October 1, 2017, (the start date of DTG implementation at AMPATH), and (3) initiated DTG on/after October 1, 2017. Pregnant WLHIV were included in the *Opt4Mamas* study if they were: (1) enrolled in HIV care at one of the five partnering HIV treatment facilities in Kisumu, Kenya, (2) newly engaging in antenatal care for this pregnancy, and (3) on ART or planning to initiate ART within 1 week. For this analysis, we included all participants who completed the COVID-19 survey in either study.

### 2.4. Data collection and management

For the *Chaguo Langu* cohort, we obtained EMR data including ART use and HIV and RH outcomes and performed a telephone survey with a subset of this cohort, purposefully selected to obtain a representative sample based on age categories (≤25, 26–35, and ≥35 years). Telephone surveys were performed by trained research assistants and conducted in the language of the participants’ choice (primarily Kiswahili). Finally, we performed qualitative in-depth interviews with a further subset of telephone survey participants, purposefully sampling from the groups of women who: (1) continued DTG, (2) switched off DTG after concerns about teratogenicity were reported, or (3) became pregnant while using DTG. Participants provided verbal consent for inclusion in the telephone surveys and written consent for inclusion in the in-depth interviews.

For the *Opt4Mamas* cohort, we enrolled 820 pregnant women and followed them prospectively every 3 months during pregnancy, at delivery, and every 3 months postpartum until 6 months postpartum, either in-person before COVID-19 or primarily over telephone after the pandemic began (August 2020 through March 2021). We also conducted qualitative in-depth interviews from December 2019 through December 2020 with a subset of participants.

For both studies, we conducted training for the in-depth interviewers on qualitative interviewing methods and ensured that interviews were conducted in participants’ preferred language (primarily Kiswahili for *Chaguo Langu* and either Kiswahili or Dholuo in *Opt4Mamas*). Interviews were audio-recorded and transcribed directly into English by the same interviewer or another member of the study staff. If the interviewers themselves were not transcribing the recording, they would review the English transcript for accuracy and any inconsistencies were resolved through conversation between study staff members.

Beginning in June 2020, questions about how the COVID-19 pandemic impacted access to care and their overall and reproductive health were added to the telephone surveys and in-depth interviews of both studies. To generate our COVID-related telephone questions, we modified a survey originally developed by investigators within the East Africa IeDEA (EA-leDEA) Consortium to assess social, economic, and health impacts of the COVID-19 outbreak on PLHIV (22). For the *Chaguo Langu* study, additional questions also addressed changes in: (1) access to FP care and (2) FP use and pregnancy intentions due to the COVID-19 pandemic. In the *Opt4Mamas* study, additional questions also addressed changes in: (1) income and (2) food insecurity due to the COVID-19 pandemic.

### 2.5. Quantitative analysis

For this analysis, we descriptively summarized the characteristics of the study participants and the proportion of respondents reporting changes in access to HIV, pregnancy, and FP care, including reasons for these changes, from both studies or the “combined cohort.” Multivariable logistic regression models were performed using the *Chaguo Langu* data to estimate the adjusted odds ratios and 95% confidence intervals for self-reported: (1) difficulty refilling medications and (2) difficulty accessing HIV care. We adjusted for the following *a priori* covariates: age (continuous), partner status (married/cohabitating vs. single), number of living children (continuous), time on ART (continuous), and HIV clinic site (discrete). We chose to limit the multivariable analysis to the *Chaguo Langu* cohort given the low reports of the outcomes of interest in the *Opt4Mamas* study. Analysis was conducted using SAS version 9.3 (SAS Institute, Cary, North Carolina, USA).

### 2.6. Qualitative analysis

Among the total 44 in-depth interviews we conducted for the *Chaguo Langu* study, only four were during the COVID-19 pandemic and thus included in this analysis. In the *Opt4Mamas* study, 23 participants underwent in-depth interviews, of which we conducted 11 during the COVID-19 pandemic and thus included in this analysis. Hence, a total of 15 in-depth interviews are included in this analysis. We uploaded the qualitative in-depth interview transcripts into NVivo (version 12.0, QRS International Pty Ltd.) for inductive coding. For the *Chaguo Langu* transcripts, team members (SAH, SK, CB, RCP) read the initial few transcripts and used their insight along with the in-depth interview guides to create the initial codebook. The *Opt4Mamas* transcripts were processed in the same manner by team members (SQ, AS, SAH). The codebooks for each study changed iteratively as the team coded the transcripts. The first 1–2 transcripts were coded in a group setting and the remainder of the transcripts were individually coded by team members. All transcripts were double-coded by CB for the *Chaguo Langu* transcripts, and SAH for the *Opt4Mamas* transcripts. Any disagreements were addressed by consensus during team meetings, which were attended by team member CB for *Chaguo Langu* sessions and RCP for all meetings. For this analysis, we included only those in-depth interviews conducted since June 2020 that explored themes related to the impact of the pandemic on RH care. We used thematic analysis techniques to organize codes into overarching domains (23, 24), and each domain was organized to include convergent and divergent sub-themes, including comparing and contrasting themes for the two cohorts and for pregnant vs. non-pregnant participants. Corresponding quotes were then extracted to illustrate the theme's depth and meaning. We report our findings for both studies below, and draw out differences between the *Chaguo Langu* and *Opt4Mamas* interview transcripts when they exist.

## 3. Results

### 3.1. Baseline characteristics

In the parent *Chaguo Langu* study, we conducted a total of 1,345 telephone surveys, of which 784 (58%) were conducted after the addition of the COVID-related questions. In the parent *Opt4Mamas* study, which included 820 participants, 618 (75%) completed surveys after the addition of the COVID-related questions. Thus, the combined cohort yielded data from 1,402 participants, with 56% of the participants from the *Chaguo Langu* study. Sociodemographic characteristics of the two participant groups individually and combined are reported in Table 1. Of note, most (83%) *Chaguo Langu* participants were currently using a FP method, however, the most common method was male condoms (46%) and few (8%) were trying to get pregnant at the time of the survey administration.

**Table 1:** Sociodemographic, HIV, and reproductive characteristics of participants of the COVID-19 quantitative survey of women living with HIV from Western Kenya (*N* = 1,402).

Characteristics	Chaguo Langu (*n* = 784)	Opt4Mamas (*n* = 618)	Combined cohort (*N* = 1,402)
**Median (IQR) or *n* (%)**			
**Age, years**	37 (28–42)	30 (25–33)	33 (27–38)
**Years of school**	8 (7–12)	12 (10–14)	10 (7–12)
Missing	259 (33.0)	1 (0.2)	260 (18.5)
**Marital status**			
Married/cohabitating	436 (55.6)	529 (85.6)	965 (68.8)
Single	338 (43.1)	88 (14.2)	426 (30.4)
Missing	10 (1.3)	1 (0.2)	11 (0.8)
**Number of living children**	2 (1–3)	2 (1–3)	2 (1–3)
**HIV clinic site**			
Moi Teaching and Referral Hospital	273 (34.8)		NA
Other AMPATH-supported sites	511 (65.2)		
Kisumu County Hospital		179 (28.9)	
Ahero County Hospital		146 (23.62)	
Nyakach Sub County Hospital		53 (8.6)	
JOOTRH*		107 (17.3)	
Lumumba Sub County Hospital		133 (21.5)	
**Time on antiretroviral therapy, years**	9 (4–12)	4 (1–6)	7 (3–11)
**Current contraceptive method use** **			
Any method	649 (82.7)	NA	NA
Condoms	364 (46.4)		
Progestin-only injectables	178 (22.7)		
Contraceptive implant	154 (19.6)		
None, natural FP, or other	117 (14.9)		
Intrauterine contraceptive device	50 (6.4)		
Sterilization	37 (4.7)		
Pills	25 (3.2)		
Missing	21 (2.7)		
**Pregnancy status**			
Currently pregnant	17 (2.2)	618 (100)	635 (45.3)
Currently trying to get pregnant	57 (7.3)		57 (4.1)
Not pregnant and not trying to get pregnant	657 (83.8)		657 (46.9)
Missing	53 (6.8)		53 (3.8)

NA, not applicable due to question not asked or unable to combine data.

* JOOTRH, Jaramogi Oginga Odinga Teaching and Referral Hospital.

** Total >100% due to potential for multiple method use.

### 3.2. Quantitative results

Our results show the following main findings, with comparison between the *Chaguo Langu* (primarily non-pregnant) and *Opt4Mamas* (all pregnant/postpartum) cohorts:

#### 3.2.1. COVID-19 pandemic impact on HIV care and treatment among WLHIV

The COVID-19 pandemic has caused greater difficulty accessing HIV care and treatment for many women, particularly those who are not pregnant and with a greater number of children, while care for pregnant women living with HIV appears to have remained largely accessible.

Most (52%) *Chaguo Langu* participants reported concern about running out of HIV medications, and many (32%) reported greater difficulty refilling medications during the COVID-19 pandemic, compared to few *Opt4Mamas* (pregnant) participants reporting the same issues (5% and <1%, respectively; Table 2). However, few participants in both groups reported skipping medications (2% in *Chaguo Langu* and 1% in *Opt4Mamas*). A minority (14%) of *Chaguo Langu* participants reported greater difficulty getting care during the COVID-19 pandemic, while few (<1%) *Opt4Mamas* participants report this same difficulty. For those who reported greater difficulty refilling medication and getting care, participants in both studies reported pharmacy stock-outs, transportation difficulties, delay in receiving services at clinic, and fear of contracting COVID as top reasons for these difficulties.

**Table 2:** Summary results of responses from participants of the COVID-19 quantitative survey of women living with HIV in Western Kenya (*N* = 1,402).

Participant responses	Chaguo Langu (*n* = 784)	Opt4Mamas (*n* = 618)	Combined cohort (*N* = 1,402)
***n* (%)**			
**Concerned about running out of medication**	406 (51.8)	18 (2.9)	424 (30.2)
**Skipped medication**	18 (2.3)	7 (1.1)	25 (1.8)
**Greater difficulty refilling medication**	249 (31.8)	1 (0.2)	250 (17.8)
**Self-reported reasons for greater difficulty refilling medication** *			
Pharmacy stock-outs	199 (78.7)	1 (0.2)	200 (14.3)
Transportation difficulties (e.g., cost, availability)	76 (30.6)	1 (0.2)	77 (5.5)
Fear of contracting COVID	16 (6.4)	0	16 (1.1)
Curfews	8 (3.2)	0	8 (0.6)
Cost	2 (0.8)	0	2 (0.1)
Other	4 (1.6)	0	4 (0.3)
**Greater difficulty getting care**	107 (13.6)	2 (0.3)	109 (7.8)
**Self-reported reasons for greater difficulty getting care** *			
Transportation difficulties	66 (61.7)	1 (0.2)	67 (4.8)
Pharmacy and clinic stock-outs	49 (45.8)	0	49 (3.5)
Delay in receiving services at clinic	6 (5.6)	0	6 (0.4)
Fear of contracting COVID	5 (4.6)	0	5 (0.4)
Curfews	4 (3.7)	0	4 (0.3)
Clinic closures	2 (1.9)	0	2 (0.1)
Other	3 (2.8)	1 (0.2)	4 (0.3)
**Greater difficulty managing family planning (FP) (including initiating, refilling, or removing methods)**	16 (2.0)	NA	NA
**Specific family planning method stock-outs**			
Progestin-only injectables	7 (43.8)	NA	NA
Contraceptive implants	4 (25.0)		
Pills	3 (18.8)		
Condoms	2 (12.5)		
**Other self-reported reasons for difficulty managing FP**			
Repeated stock-out related difficulties	11 (68.7)	NA	NA
Cost	2 (12.5)		
Mobile clinic closures	1 (6.3)		
Transportation difficulties	1 (6.3)		
**Change in likelihood of using family planning**			
No difference	743 (94.8)	NA	NA
Less likely to use family planning	22 (2.8)		
More likely to use family planning	18 (2.3)		
**Change in desire to get pregnant soon**			
No difference	741 (94.5)	NA	NA
Less likely to want to get pregnant soon	39 (5.0)		
More likely to want to get pregnant soon	4 (0.5)		
**Source of food during outbreak**			
Stores or market	NA	563 (91.1)	NA
Own farm		265 (42.9)	
Relative or friend's farm		96 (15.5)	

NA, not applicable due to question not asked.

* Total >100% due to potential for multiple response options.

Logistic regression models performed with the *Chaguo Langu* data showed that there was a trend toward women with more children being *more* likely to report difficulty refilling medication (aOR = 1.13, 95% CI: 1.00–1.28), while older women were *less* likely to report difficulty refilling medication (aOR = 0.95, 95% CI: 0.92–0.98). None of the factors assessed in logistic regression models were associated with self-reported greater difficulty accessing care since the beginning of the COVID-19 pandemic.

#### 3.2.2. COVID-19 impact on family planning services

Overall, among non-pregnant WLHIV of reproductive age in our cohort, family planning access has largely remained stable during the COVID-19 pandemic. Among the *Chaguo Langu* participants, few (2%) reported difficulty managing family planning, including initiating, refilling, or removing methods (Table 2). Of those who did report difficulty managing FP, all reported stock-outs as the reason, with stock-outs affecting a range of methods including injectables, implants, pills, and condoms. Secondary reasons for difficulty managing FP included cost, mobile clinic closures, and transportation difficulties.

#### 3.2.3. COVID-19 impact on pregnancy intentions

WLHIV did not report changes in family planning use and pregnancy intentions during the COVID-19 pandemic, however most were using a less effective method of contraception.

Among the *Chaguo Langu* participants, most (84%) were not currently pregnant and not trying to get pregnant. The majority (95%) reported no change in their likelihood of using family planning since the beginning of the COVID-19 pandemic; 3% reported being less likely to use FP and 2% reported being more likely to use FP. Similarly, 95% reported no change in their desire to get pregnant soon, while 5% reported being less likely to want to get pregnant since the beginning of the COVID-19 pandemic. Most (83%) participants reported using some method of contraception, however, the most commonly used method (46%) was condoms, and only about half collectively reported using a highly effective method (23% injectables, 20% implants, 6% IUCDs, and 5% permanent sterilization).

### 3.3. Qualitative results

Among the total 44 in-depth interviews a total of 15 in-depth interviews are included in this analysis from both studies. Baseline characteristics of the in-depth interview participants are described in Table 3.

**Table 3:** Sociodemographic characteristics of participants of in-depth interviews of women living with HIV in Western Kenya (*N* = 15).

Characteristic	Chaguo Langu (*n* = 4)	Opt4Mamas (*n* = 11)	Combined cohort (*N* = 15)
**Median (IQR) or *n* (%)**			
**Age**			
<20	0	0	0
20–30	1 (25)	6 (54)	7 (47)
>30	3 (75)	5 (45)	8 (53)
**Occupation before pandemic**			
Housewife	NA	4 (36)	NA
Vegetable vendor		2 (18)	
Farmer		1 (9)	
Teacher		1 (9)	
None		3 (27)	
**Number of living children**			
0	0	1 (9)	1 (7)
1–3	4 (100)	10 (91)	14 (93)
**Antiretroviral therapy base**			
Dolutegravir	2 (50)	11 (100)	13 (87)
Other antiretroviral	2 (50)	0	2 (13)
**Contraceptive method use (current or most recent method used if pregnant)**			
Condoms	0	1 (9)	1 (7)
Progestin-only injectables	1 (25)	3 (27)	4 (27)
Contraceptive implant	2 (50)	1 (9)	3 (20)
None, natural FP, or other	1 (25)	5 (45)	6 (40)
Pills	0	1 (9)	1 (7)
Intrauterine contraceptive device	0	0	0
Sterilization	0	0	0
**Transport between house and clinic**			
Walking	NA	1 (9)	NA
Minibus (*Matatu*)		2 (18)	
Motor Bike (*Boda boda*)		5 (45)	
*Tuk Tuk*		2 (18)	
Buses		1 (9)	
**Pregnancy status**			
Currently pregnant or within 6 months postpartum	0	11 (100)	11 (73)
Currently trying to get pregnant	0	0	0
Not pregnancy and not trying to get pregnant	4 (100)	0	4 (27)

NA, not applicable due to question not asked or unable to combine data.

In our qualitative analysis we focused on the potential influences of the COVID-19 pandemic on access to HIV and reproductive healthcare and pregnancy intentions and family planning use. Three major themes emerged: (1) organizational and economic implications of the COVID-19 pandemic, (2) influence of the COVID-19 pandemic on pregnancy intentions and prevention, and (3) fear of contracting COVID-19. These major themes illustrate underlying drivers for the findings of the quantitative analysis. We discuss these themes in further detail below with supportive quotes, see Supplementary Material for more example quotes.

#### 3.3.1. Adverse organizational and economic implications of the COVID-19 pandemic

##### 3.3.1.1. Transportation difficulties due to the COVID-19 pandemic

*“The only difficulty is the increase in cost of transportation. Where we used to be charged Ksh 100, currently it is Ksh 200, where we were charged Ksh 50 we currently pay Ksh 80 or Ksh 100. So, we are paying twice what we used to pay before.”* (36 years old, not using FP, using non-DTG ART)

*“I am just okay. I have been attending my clinic. But the challenge is that I sometimes don't have money for transport and so I have to push my dates.”* (28 years old, implant user, using non-DTG ART)

Transportation problems were identified as a cause for having difficulty managing FP and refilling medicine in our quantitative analysis, which was also a recurring theme among in-depth interview participants. When prompted to discuss reasons for difficulty accessing care and difficulty refilling medications, the discussion often centered around difficulties with accessing transportation. Participants discussed that public transportation is the most common mode of accessing clinic and pharmacy care and since the pandemic began the cost of transport significantly increased and its availability decreased. One participant noted that the cost of bus fare had doubled since the pandemic began and another participant explicitly reported delaying care-seeking due to lacking funds for transportation.

##### 3.3.1.2. Healthcare and supply chain disruptions due to the COVID-19 pandemic

*“I had concerns since no airplanes are flying in or out of our country at the moment. That's so dangerous and one must have concerns because if you are told that the medications are no longer available, then what else would you do? The medication is life.”* (34 years old, injectable and female condom user, using DTG-based ART)

Participants reported continued functioning of and general access to the clinics where they receive care, which include HIV, pregnancy, and family planning services. However, they reported significant concern about running out of medications due to issues with medication supply chains and expressed significant fear about the implications of losing access to life-saving ARVs. Fear of stock-outs and among those who reported difficulty refilling medication in our quantitative analysis, accessing care, or managing FP, many cited pharmacy and clinic stock-outs as leading reasons for their difficulties. Other participants reported additional care disruptions, such as fewer clinic staff providing care, which resulted in longer wait times and less time with the providers. While several participants expressed not being concerned about drug stock-outs, one participant stated that they are concerned because they realize ARVs are essential to the survival of people living with HIV.

##### 3.3.1.3. Loss of income/employment

*“Income is limited because I don't go to work; my husband is the one working…I don't have any work and so I depend on him for everything.”* (31-year-old, pregnant, using non-DTG ART)

“*It has affected me because I can no longer do my business.”* (23-year-old, pregnant, using non-DTG ART)

The survey results indicated that the cost of FP methods was a barrier to managing FP during the pandemic. Additionally, interview participants reported that pandemic-related loss of employment had wide-ranging implications and made it difficult to pay for healthcare-related needs, such as transportation to healthcare facilities, family planning methods, and food. When asked about challenges they encountered during the pandemic, several participants instantly mentioned financial concerns, stating that this was a more pressing issue in their life than access to HIV treatment.

##### 3.3.1.4. Food insecurity

*“I have experienced only one challenge. Since the onset of the Corona pandemic worldwide, you find that sometimes I lack proper food and yet I'm on medication. When you go for more medication, the doctor advises you to take proper meals alongside the medication but sometimes you can't afford it. This forces you to borrow flour from your neighbor so that you can at least make some porridge and take it the whole day; you just have to pray to God. I have experienced this.”* (26-year-old, pregnant, using non-DTG ART)

Interview participants reported lacking access to food due to loss of income/employment and the need to avoid crowded market spaces where food is purchased. One participant in particular reported that she was told to stay away from the market due to being at high-risk for complications of COVID due to both her pregnancy and HIV status. Other participants mentioned asking neighbors for staple foods, such as flour to make porridge, that will sustain them for long periods of time. Lack of food was also reported as a barrier to taking their medications, since providers urge patients to take ARVs with proper meals.

#### 3.3.2. Increased importance of family planning use to prevent pregnancy during the COVID-19 pandemic

*“Life has become hard, the economic is getting worse; everything is getting worse. So if you decide to conceive, then life will be harder.”* (34 years old, injectable and female condom user, using DTG-based ART)

*“Just that at the moment, people are at home since there are no jobs. There is no giving birth with this hard economy.”* (36 years old, not using FP, using non-DTG ART)

Interview participants overwhelmingly stressed how important it was for them to prevent pregnancy during the COVID-19 pandemic. Specifically, they emphasized the importance of using FP to protect themselves from unintended pregnancy due to new or heightened economic difficulties caused by the pandemic, this was also reflected in our quantitative analysis. Barriers to managing FP reported in the interviews included method stock-outs and reduced access to facilities for FP care due to clinic closures and clinic staff reductions. Many reported taking precautionary steps like stockpiling condoms whenever they attend the clinic.

#### 3.3.3. Fear of contracting COVID-19, particularly for people living with HIV

*“They were saying people who are HIV positive were mostly dying from the disease. It really made me scared. So, I was staying indoors, and I could avoid going to the shops and also was avoiding people. Yes, we could meet but we kept distance but not staying close to each other.”* (36 years old, not using FP, using non-DTG ART)

Interview participants, similar to the telephone survey respondents, discussed concerns about contracting COVID-19, particularly related to the potential additional risks of infection facing PLHIV and particularly pregnant WLHIV. One participant reported fear of death from COVID-19 because she believed a majority of people dying from COVID-19 were those living with HIV. Participants reported that their HIV status affected how they responded to the pandemic, and they were highly aware of how actions that increased their risk of contracting COVID-19 may impact their health. To mitigate these risks, participants reported increasing hand-washing and house-cleaning, as well as social behaviors, such as social-distancing, staying indoors/avoiding crowded places, and avoiding handshakes.

## 4. Discussion

Our mixed methods analysis show how varying groups of WLHIV in western Kenya are experiencing the impact of the COVID-19 pandemic differently for HIV and reproductive health care. We found that a substantial number of WLHIV report concerns about running out of medications, difficulty refilling medications, and accessing HIV services since the start of the pandemic. While most pregnant WLHIV in our sample indicated minimal concerns or disruptions in their access to care, many non-pregnant WLHIV reported concerns about managing medication refills and access to HIV care, but few concerns about managing family planning. However, nearly all WLHIV reported concerns about transportation issues and financial hardships brought on or exacerbated by the pandemic. Our qualitative research found that the participants’ financial concerns had a significant influence on their perception of the importance of family planning in preventing an unwanted pregnancy. This is consistent with evidence that globally women have been disproportionately impacted by the COVID-19 pandemic, particularly related to the loss of income coupled with increasing household demands (9, 25).

In both quantitative and qualitative answers, participants’ self-reported reasons for difficulties in accessing care are complex and include both structural and individual factors. Policies enacted during the pandemic that aimed to prevent the spread of COVID-19 had unintended consequences such as mass unemployment, particularly in the informal work sector, and participants commonly reported losing a reliable source of income to support their families, thus impacting their ability to pay for rising transportation costs and other expenses related to medical care. Like many limited income countries, Kenya continues to lag behind in protecting its population from the financial risks connected with illness and healthcare seeking behavior (26, 27). A study looking into the out-of-pocket (OOP) expenditures of the Kenyan healthcare system, found that about 1.1 million people are forced into poverty as a result of OOP payments (27). To protect the most vulnerable population groups from the high costs of sickness, more effort is necessary in limited income countries like Kenya (27–29). In 2004, the Kenyan government modified its national insurance program and implemented the National Hospital Insurance Fund (NHIF) cover to facilitate cheaper access to health care in the country, in accordance with the World Health Organization's Universal health coverage objectives (30). However, from its inception, the NHIF has failed to meet the expanding health care demands of Kenya's rising population (30). According to the Ministry of Health (MoH Kenya) (2019), just 11% of Kenyans are covered by this insurance program, leaving 89% of the population uninsured (30). Since the majority of Kenyans get health care *via* out-of-pocket payment, many would avoid coming to the hospital until they are in the latter stages of an illness (30). Given the characteristics of COVID-19, such a delay might result in the disease's devastating spread. Additionally, food insecurity was not only exacerbated by job losses and income reductions, but also by limited access to food markets. This hardship especially affects PLHIV, whose adherence to antiretroviral treatment may be worsened by food insecurity, with impacts on health outcomes (31).

Our results highlight the importance of public transportation in access to care and the unintended impact of public health interventions that have aimed to limit the spread of COVID-19 but have unintentionally increased transportation costs and reduced availability. During the pandemic, transportation providers raised their fares to compensate for the additional expenses associated with lower capacity and sanitation services (32). A study conducted in Kenya regarding COVID-19 implications, has a participant that articulated that they would now have to pay double the fare to compensate for the vacant seats during the pandemic (32). The long-term health implications of such restrictions are currently unknown (19, 33, 34). Across both our quantitative and qualitative data, our evidence highlights the concern that rising costs and decreased access to transportation due to the pandemic creates a significant barrier to accessing healthcare services among WLHIV. Notably, transportation cost increases may be especially difficult for women with significant household responsibilities, as our logistic regression models showed that women with more children were more likely to report difficulty refilling medication. There is a need to mitigate the unintended impacts of public health measures on WLHIV and other vulnerable populations facing economic burdens, food insecurity, and potential disruptions to lifesaving medications and treatment.

Our results also document the impact of stock-outs. Supply chain disruptions have hampered access to pharmaceuticals and food and nutrition products, particularly in resource-limited settings (3, 35). WLHIV we interviewed are acutely aware of the dangers they face as a result of both ARV and family planning stock-outs and are taking actions to avoid the potentially devastating results of losing access to this care. Importantly, for WLHIV receiving care at the sites we studied, stock-outs have had great effect on their access to medication and family planning methods. A shortage of antiretroviral therapy (ART) in Kenya during the pandemic resulted in the development of resistance to particular treatments, the occurrence of opportunistic infections, and an increase in HIV-related mortality overall (36, 37). According to Kenyan news sources, the HIV drug shortage was primarily caused by the COVID-19 pandemic and the stockpiling in customs warehouses caused by the increase in government taxes (38). However, in many settings preemptive measures were taken by the Ministry of Health and care programs to minimize the impact of these shortages on clinical care, thus many participants did not report being affected by these shortages. There is a need to address global supply chain challenges that result in disrupted access to essential medications and other healthcare commodities.

Interestingly, the ability for pregnant WLHIV in the Opt4Mamas cohort to access care seems largely uninterrupted. This may be due to differences within the care programs, geographical milieu, or demographics of the cohorts, for example the Opt4Mamas cohort had greater educational attainment and were more likely to be married. Additionally, it is possible that pregnant and postpartum women may have received priority for medication and medical care in general, therefore avoiding the challenges faced by non-pregnant women. Similarly, WLHIV reported preserved ability to manage their family planning. These findings are similar to some reports of overall preserved pregnancy care access (39), but differs from other reports of large-scale interruptions in family planning care, leading to unintended pregnancy (12). However, most study participants identified male condoms as their leading family planning method, which may be more accessible than other methods that require healthcare providers, facilities, and more complex supply chains. Additionally, this cohort included few adolescents and may not be representative of other groups who faced significant challenges accessing FP in general and especially during the pandemic. Some participants did report difficulty managing their family planning, including due to method stock-outs of commonly used injectables and implants. Most quantitative survey participants reported continued and strong desire to prevent pregnancy, but no clear effect of the pandemic on these decisions. However, participants in the in-depth interviews universally spoke about how the pandemic has caused an urgent need to protect themselves from unintended pregnancy due to pandemic-related economic hardships that would worsen with the addition of another child. In light of this, the reliance on condoms, one of the least effective contraceptive methods, as their main method of pregnancy prevention highlights the importance of counseling and shared decision-making among providers and WLHIV regarding the risks of pregnancy occurring with less effective methods and the availability of more effective methods. Given the vital need to prevent pregnancy to avoid economic hardship (40), any difficulty accessing family planning methods could have significant impacts on the lives of WLHIV and their families. Additionally, effective family planning use to avoid unintended pregnancy is one of the four pillars of preventing mother-to-child transmission of HIV, and focus should remain on how to best achieve these goals for WLHIV during the ongoing pandemic (41).

Similar to other studies, we found that fear of contracting COVID-19 may be particularly salient for WLHIV due to knowledge that HIV increases the risk of COVID-19 infection and disease severity (31, 33). Additionally, there are significant adverse outcomes associated with COVID-19 infection during pregnancy (42, 43). This fear may significantly affect care-seeking behavior, especially when doing so puts them in situations at high risk for acquiring infection, like crowded public transportation or long clinic queues. Though pregnant WLHIV were prioritized for care during the COVID-19 pandemic and facilities maintained their usual antenatal care schedule (44), it is not clear how many women adhered to this schedule given their potential fears of contracting COVID-19. While HIV care programs are adapting by increasing telehealth and virtual care platforms, community-based ARV delivery, and identifying particularly vulnerable individuals and populations for intensified outreach strategies (45, 46), it remains unclear what the ultimate downstream consequences may be for PLHIV experiencing reduced access to care.

While our findings provide significant insight on the various challenges faced by WLHIV as a result of the COVID-19 pandemic, our study has a few limitations. Unfortunately, we were not able to connect the participants’ self-reported outcomes to clinical outcomes such as viral suppression and unintended pregnancy rates. Considering the COVID-19 pandemic added complexity to conducting a large number of qualitative interviews, we may not have captured all perspectives of WLHIV during this time period. However, we interviewed a number of women, both pregnant and non-pregnant, with the hope of capturing a range of viewpoints. Our interviews were also conducted during the first phase/peak of COVID-19 infections in Kenya, and thus, relatively early; it is possible that the same surveys or interviews administered now, 2 years into the global pandemic, would elicit different responses. In addition, we integrated data from two distinct studies with comparable participant groups, whose themes frequently overlapped but were otherwise distinct. Furthermore, we acknowledge that cohort grouping and generalizing the perspectives of four participants from a single study who may not have been representative of the wider cohort of participants for Chaguo Langu led to themes that were congruent with those of the Opt4mamas study. Despite these limitations, we believe our work is the most comprehensive, utilizing both quantitative and qualitative data collection methods, to date on the impact of the COVID-19 pandemic on access to care for WLHIV in western Kenya.

## 5. Conclusion

As the COVID-19 pandemic continues, it will be important for HIV, RH, and COVID-19 programs and policymakers to monitor the changing landscape and impact the pandemic has on access to care for WLHIV. While specific concerns and challenges varied among pregnant vs. non-pregnant WLHIV, nearly all reported concerns about transportation issues and financial hardships brought on by the pandemic. Innovative ways of maintaining and improving access to care, medication refills, and family planning initiation and continuation will be necessary to ensure that the HIV and reproductive health outcomes for WLHIV and their children do not worsen during the COVID-19 pandemic.

## Data Availability

The raw data supporting the conclusions of this article will be made available by the authors, without undue reservation.
