# Supplementary material for: Impacts of the COVID-19 pandemic on access to HIV and reproductive health care among women living with HIV (WLHIV) in Western Kenya: A mixed methods analysis
Source: Front Glob Womens Health. 2022 Dec 12;3:943641. doi: 10.3389/fgwh.2022.943641 (PMC9790904; doi:10.3389/fgwh.2022.943641)
Supplement: Supplementary file 1 [file Table1.docx]

**Supplementary Table.** Main themes, subthemes, and example quotes from qualitative analysis of in-depth interviews of women living with HIV in western Kenya during the COVID-19 pandemic. (N=15)

| **Main theme** | **Subtheme** | **Example quotes** |
| --- | --- | --- |
| **Organizational and economic implications of the COVID-19 pandemic** | Stability of access to care | I: And in general, when it comes to Maternal Child Health clinic services, has there been any change since the outbreak?  R: None. We are being attended just like before the outbreak. *(28 years old, non-pregnant, implant user)*  I: Has the Coronavirus pandemic affected your daily life and adherence to HIV medications? R: The Coronavirus has not affected my life as such because we are living with it; it hasn’t prevented me from taking my medications either. We just follow the laid down guidelines while coming for medications. (*37-year-old, pregnant, using non-DTG ART*)  I: Has the Coronavirus pandemic affected your ability to come to the clinic for viral load testing? R: No, Corona has not affected it any way. However, sometimes I can lack transport, but I can borrow from someone and repay them once I’m able to. I have not missed any of my clinic appointments. (*27-year-old, pregnant, using non-DTG ART)*  *Responses when asked: “Have you had any concerns about running out of your medication since the outbreak began?”*  R: I have never thought of running out of medications because I have been going for the check up at the clinic.  I: So, you’ve had enough supply of your medications?  R: Yes.  I: Have you had greater difficulties than usual in getting HIV care and other medical care during this time?  R: No, I haven’t had any difficulty. (*39 years old, non-pregnant, using DTG-based ART)*  R: I had no concerns of running out of medications since we have been going to the clinic and we are served well as usual.  I: And have you had any difficulties in managing your family planning since the outbreak began?  R: I haven’t had any difficulty because whenever I go to the facility, I pick a full box of condoms. (*36 years old, non-pregnant, using non-DTG ART)* |
|  | Rising cost of transportation impacts access to care | I: Have you skipped any medication doses because you were concerned about running out of medications?  R: Even if it was going to run out, I just live near the clinic. Although the cost of transportation had risen, but I have never missed my clinics.  I: How has the novel Coronavirus pandemic impacted your overall well-being and how you take your ARVs?  R: It impacted on me sometimes back. The public service transport emphasized on strict measures of social distancing, so I had no option but to use a lot of fare and sometimes used motorbike to the facility, so the fare was hiked higher. (*30-year-old, pregnant*, *using non-DTG ART)*  I: Has the Coronavirus pandemic affected your ability to come to the clinic for viral load testing? R: No, Corona has not affected it any way. However, sometimes I can lack transport, but I can borrow from someone and repay them once I’m able to. I have not missed any of my clinic appointment. (*25-year-old, pregnant,* *using non-DTG ART)*  I: How has COVID-19 affected your adherence to HIV medication?  R: It hasn’t affected me in any way except that bus fare went up because I chose this clinic. For other people, you find that there is a lockdown and maybe one takes medication in Kilifi yet he is in Mombasa. I had a relative who was affected by this but he got help from the nearest facility.  I: So, you have been getting your medications well irrespective of COVID-19?  R: Yeah, it has all been okay. (37*-year-old, pregnant,* *using non-DTG ART)*  *Responses to: “Have you had greater difficulties than usual in getting HIV care and other medical care during this time?”*    R: The only difficulty is the increase in cost of transportation. Where we used to be charged Ksh 100, currently it is Ksh 200, where we were charged Ksh 50 we currently pay Ksh 80 or Ksh 100. So, we are paying twice what we used to pay before. (*36 years old, non-pregnant, using non-DTG ART*)  R: None. I am just okay. I have been attending my clinic. But the challenge is that I sometimes don’t have money for transport and so I have to push my dates.  I: And do you think there has been any changes when it comes to accessing Maternal Child Health clinic services since the outbreaks of Coronavirus began?  R: Yes. Especially if you are not wearing a face mask, then you wouldn’t be attended by the doctor. Because you might have been infected with the disease and then you end up transmitting to the doctor. *(28 years old, non-pregnant, using non-DTG ART)* |
|  | Supply chain disruptions | I: Have you had any concerns since the Coronavirus outbreak began?  R: Yes. I had concerns since no airplanes are flying in or out of our country at the moment. That’s so dangerous and one must have concerns because if you are told that the medications are no longer available, then what else would you do? The medication is life. (*34 years old, non-pregnant, using DTG-based ART*) |
|  | Loss of employment and source of income | I: Since the outbreak of Coronavirus, is there a way it has affected you when it comes to accessing your drugs? R: No. it hasn’t affected me.  I: Okay. And in terms of livelihood, has it affected you in anyway? R: Yes. Because there are no jobs. I: So, you were working before and right now you are not working anymore? R: Yes. I: What was the reason? R: I just left mine but for my spouse, he lost his job. I: What the reason why he lost his job? R: When the outbreak started, he stopped going to work (*23-year-old, pregnant, using non-DTG ART)*  I: Has the Coronavirus pandemic affected your adherence to ARVs in any way?  R: Income is limited because I don’t go to work; my husband is the one working…I don’t have any work and so I depend on him for everything (*31-year-old, pregnant, using non-DTG ART)*  I: The Coronavirus pandemic, has it impacted your HIV care and ability to take your ARVs or impacted your general well-being? R: It has affected me because I can no longer do my business (*23-year-old, pregnant, using non-DTG ART)*  I: How has the novel Coronavirus pandemic impacted your HIV care and ability to take your ARVs?  R: No, it hasn’t affected me that much maybe in terms of financial status  I: Were you able to pick your drugs as scheduled?  R: Yeah (*22-year-old, pregnant, using non-DTG ART)* |
|  | Food insecurity | I: As a pregnant woman living with HIV, what challenges have you experienced in your adherence journey as compared to before you became pregnant.. regarding your adherence to medication while you are pregnant, what specific challenges have you experienced? R: I have experienced only one challenge. Since the onset of the Corona pandemic worldwide, you find that sometimes I lack proper food and yet I’m on medication. When you go for more medication, the doctor advises you to take proper meals alongside the medication but sometimes you can’t afford it. This forces you to borrow flour from your neighbor so that you can at least make some porridge and take it the whole day; you just have to pray to God. I have experienced this. I: You have experienced this? R: Yes. I: So, you are saying that at times you lack food to take so that medications can work well in your body? R: Yes. (*26-year-old, pregnant,* *using non-DTG ART)*  I: How has the COVID-19 pandemic affected you personally with relation to picking your ARVs? How has it affected you? Has it brought you some challenges in that there is difference on how you take your drugs? R: The challenge that it has brought is financial problem. We used to do our business and we managed to get something to put food on the table. But when COVID-19 was reported things were tough and we were put under lockdown. Doing business became extremely difficult to the extent that we find it hard to do business and put food on the table. It was a challenge and we just remained indoors. In my case, it came in when I was pregnant and they could easily tell me, ‘Eeeeeh you can easily get infected with this disease, move out of here.’ Even in the market. I found it too rough and just remained in my house praying for God to remember us because it is extremely hard.  I: So, you are taking your drugs or which challenges is it contributing with your drugs? R: I was just taking my drugs. The challenge I am facing is getting something to eat… It is a challenge getting something to eat because there is no way. *(35-year-old, pregnant, using non-DTG ART)* |
| **Importance of family planning use to avoid unintended pregnancy that would exacerbate economic hardship faced during the COVID-19 pandemic** |  | *Responses to: “If you compare before the Coronavirus outbreak and after the outbreak, has the outbreak made you more or less likely to use family planning compared to before the outbreak?*  R: It has made me more likely to use FP.  I: Could you tell me more about that?  R: Why I have said that … because now with this virus you don’t know when it will end and having another child with this situation the way the economy is, it’s so hard. (*39 years old, non-pregnant, using DTG-based ART*)  R: I really have to use family planning for now; it is so dangerous, and I have to protect myself.  I: Protect yourself from?  R: Protection from unplanned pregnancies since life has become harder; right now you can’t go knocking on people’s doors, you can’t walk around. So you have to protect yourself.  I: So the outbreak has made you more likely to use family planning?  R: Yes.  I: Has the Coronavirus outbreak made you more or less likely to want to get pregnant soon compared to before the outbreak?  R: At the moment I wouldn’t really want to get pregnant. It’s a challenge.  I: It’s a challenge in what ways?  R: Life has become hard, the economic is getting worse; everything is getting worse. So if you decide to conceive, then life will be harder. *(34 years old, non-pregnant, using DTG-based ART)*  R: I think I am just okay. I don’t see it being more likely. Just that at the moment, people are at home since there are no jobs. Where will one get the money to replace the one you have used when traveling and yet there are no jobs? There is no giving birth with this hard economy. *(36 years old, non-pregnant, using non-DTG ART)* |
| **Fear of contracting COVID-19 due to increased risk of COVID-19 for people living with HIV** |  | I: What have you heard about Coronavirus or COVID-19?  R: I have heard that’s it’s so dangerous especially to people like us  I: What do you mean when you say people like us?  R: People like us have a big problem in case one contract the disease, then it might be worse.  I: Okay. Apart from that, what else have you heard about Coronavirus?  R: That’s all that I have heard. So, we really have to take care of ourselves.  I: In what way?  R: Covering our mouths, washing hands and making sure everything is clean in where we stay. (*34 years old, non-pregnant, using DTG-based ART)*  I: Have you had any concerns since the outbreak of Coronavirus began?  R: Yes. They were saying people who are HIV positive were mostly dying from the disease. It really made me scared. So, I was staying indoors, and I could avoid going to the shops and also was avoiding people. Yes, we could meet but we kept distance but not staying close to each other. (*36 years old, non-pregnant, using non-DTG ART*)  I: What have you heard about Coronavirus or COVID-19?  R: I have heard people saying that we should wear masks and wash hands. And the symptoms of the disease are flu like, sneezing, and chest problems. And what it really scares me is that if one is HIV positive, then you have a high chance of contracting the disease. So, we been doing what we are told; washing hands and avoiding handshakes. (*28 years old, non-pregnant, using non-DTG ART)* |

FP = family planning, DTG = dolutegravir, ART = antiretroviral therapy
